# Differential associations of IgG-aFcεRI and IgG-aIgE autoantibodies with clinical phenotypes of type IIb chronic spontaneous urticaria^☆^

**DOI:** 10.1016/j.waojou.2026.101411

**Published:** 2026-05-30

**Authors:** Lāsma Lapiņa, Stefan Frischbutter, Jween Mohammad, Guna Ziedone, Nataļja Kurjāne

**Affiliations:** a Department of Biology and Microbiology, Riga Stradiņš University, Riga, Latvia; b Pauls Stradiņš Clinical University Hospital, Center for Clinical Immunology and Allergology, Riga, Latvia; c Allergic Diseases Diagnosis and Treatment Center, Riga, Latvia; d Institute of Allergology, Charité - Universitätsmedizin Berlin, Corporate Member of Freie Universität Berlin and Humboldt-Universität zu Berlin, Berlin, Germany; e Fraunhofer Institute for Translational Medicine and Pharmacology ITMP, Immunology and Allergology, Berlin, Germany

**Keywords:** Chronic urticaria, Angioedema, Autoantibodies, Biomarkers, Phenotype

## Abstract

**Background:**

Chronic spontaneous urticaria (CSU) is a common mast cell-driven skin disorder characterized by recurrent wheals and/or angioedema lasting more than 6 weeks. A subset of patients shows type IIb autoimmune features, including IgG autoantibodies against FcεRI or IgE, which have been associated with more severe and treatment-refractory disease.

**Objective:**

This study aimed to explore the associations of IgG-aFcεRI and IgG-aIgE autoantibodies with clinical and laboratory features of CSU and to assess whether these autoantibody patterns are linked to distinct clinical phenotypes.

**Methods:**

Adult patients with CSU underwent standardized clinical evaluation and completed validated patient-reported outcome measures (UCT, UAS7, CU-Q2oL, AECT, AAS, AE-QoL, and VAS). Venous blood samples were collected and analyzed for HSP70, anti-HSP70, and IgG autoantibodies against FcεRI and IgE using ELISA. Additional laboratory data were obtained from the national DataMed system. Statistical analyses were performed using Jamovi v2.7.5, with p < 0.05 considered statistically significant.

**Results:**

The study included 205 adults (77.6% female; mean age 42.8 years), of whom 199 were tested for IgG-aFcεRI and IgG-aIgE. Higher IgG-aIgE levels were observed in patients with angioedema (either angioedema alone or urticaria with angioedema), as well as in those reporting accompanying symptoms, receiving omalizumab treatment, living in rural areas, and experiencing predominantly nighttime symptoms. In contrast, higher IgG-aFcεRI levels were associated with wheal-predominant disease and anti-TPO positivity. IgG-aFcεRI levels correlated negatively with IgG-aIgE, neutrophil count, and total IgE, and positively with total IgG.

**Conclusion:**

Higher IgG-aIgE levels were associated with angioedema, accompanying symptoms, omalizumab use, rural residence, and nighttime symptom patterns, suggesting an association with distinct clinical features of CSU. In contrast, higher IgG-aFcεRI levels were associated with wheal-predominant disease and anti-TPO positivity.

## Introduction

Chronic spontaneous urticaria (CSU) is a common and burdensome mast cell-driven skin disorder, defined by the recurrent occurrence of intensely pruritic wheals, angioedema, or both, on a daily, near daily, or intermittent basis for more than 6 weeks.^1^^,^^2^ The mechanisms underlying mast cell degranulation differ among patient subgroups and are commonly categorized into 2 main immunological endotypes.

• Type I (autoallergic) CSU is mediated by IgE antibodies directed against self-antigens, such as IL-24, thyroid peroxidase (TPO), double-stranded DNA (dsDNA), and other autoantigens. These IgE autoantibodies bind to the high-affinity IgE receptor (FcεRI) on mast cells and basophils. When the corresponding antigen is present, receptor cross-linking occurs, triggering mast cell degranulation and the release of histamine, proteases, cytokines, chemokines, and other pro-inflammatory mediators, which collectively contribute to the development of wheals and/or angioedema.^3^, ^4^, ^5^, ^6^, ^7^
• Type IIb (autoimmune) CSU involves IgG antibodies targeting either FcεRI or IgE itself. These antibodies can directly activate mast cells or engage the complement system, producing C5a, which further stimulates mast cells and basophils. IgG1 and IgG3 subtypes are particularly implicated. Patients with type IIb CSU often present with more severe, treatment-resistant disease and show a higher prevalence of autoimmune comorbidities.^4^^,^^6^^,^^8^, ^9^, ^10^

CSU is increasingly recognized as a heterogeneous disease with multiple underlying immunological mechanisms.^2^^,^^8^ While type I autoallergic CSU is thought to account for the majority of cases, a smaller subset of patients exhibits features consistent with type IIb autoimmune mechanisms, which have been associated with higher disease activity, lower total IgE levels, and reduced response to antihistamines.^8^^,^^11^^,^^12^ Identifying clinical and laboratory characteristics associated with these autoimmune features remains an important goal in improving disease stratification and guiding individualized treatment approaches.

Our study aimed to investigate whether elevated levels of IgG autoantibodies against FcεRI or IgE, which have been associated with type IIb autoimmune mechanisms in CSU and mast cell activation,^8^^,^^11^^,^^12^ are linked to specific clinical and laboratory characteristics that may assist in identifying autoimmune-related CSU phenotypes.

Identifying biomarkers associated with autoimmune mechanisms in CSU may be clinically relevant, as several emerging therapies target mast cell activation pathways beyond IgE-mediated mechanisms, including BTK inhibitors, anti-KIT antibodies, and other targeted immunomodulatory treatments.^13^, ^14^, ^15^, ^16^, ^17^, ^18^

## Methods

### Patients

Adult patients (≥18 years) diagnosed with CSU between January 2016 and May 2023 were identified through the hospital electronic medical records system and from a specialized center for allergic diseases. All participants provided written informed consent prior to inclusion in the study. The study protocol was approved by the relevant ethics committee (Approval No. 2-PĒK-4/68/2023).

A total of 205 patients with CSU were included in the study cohort. Serum samples for the measurement of IgG autoantibodies against FcεRI and IgE were available for 199 patients, who were therefore included in the autoantibody analyses.

### Data acquisition

During scheduled study visits, participants completed a series of standardized and validated patient-reported outcome measures, including the Chronic Urticaria Quality of Life Questionnaire (CU-Q2oL), Urticaria Control Test (UCT), Urticaria Activity Score over 7 days (UAS7), and Urticaria Severity Score (USS). Angioedema-specific assessments included the Angioedema Quality of Life Questionnaire (AE-QoL), Angioedema Activity Score (AAS), Angioedema Control Test (AECT), and the Visual Analogue Scale (VAS).

Anthropometric parameters, educational background, occupational status, smoking habits, comorbidities, and detailed family and personal medical histories related to allergic, autoimmune, oncological, and other conditions were systematically recorded. Disease-specific parameters included CSU duration, anatomical distribution of pruritus and wheals, circadian patterns of symptom occurrence, potential triggers, current and previous treatments, and treatment response. Disease intensity was additionally assessed using the Visual Analogue Scale (VAS).

Participants were also assessed for accompanying systemic symptoms. These included headache, joint pain, rhinitis, cough, gastrointestinal symptoms (nausea, vomiting, abdominal pain, or diarrhea), fatigue, palpitations, subfebrile temperature, anxiety, dizziness, shortness of breath, skin burning or cold sensations, burning sensation in the legs, and muscle pain.

Venous blood samples were collected under standard aseptic conditions. For serum preparation, blood was drawn into serum-separating tubes and allowed to clot at room temperature for 30 min. Samples were centrifuged at 1500 × *g* for 10 min, and the resulting serum was aliquoted into cryovials. For plasma preparation, blood was collected into EDTA tubes, and centrifuged at 1500 × *g* for 10 min. The plasma supernatant was transferred into cryovials. All serum and plasma aliquots were immediately stored at −80°C until analysis.

Patient interviews and biological sample collection were conducted between October 2022 and May 2023.

### Laboratory data acquisition

Laboratory test results were retrieved from the national electronic medical information system, which integrates laboratory and imaging data from multiple certified laboratories across the country, ensuring standardized and reliable measurements. The following parameters were extracted: leukocyte subpopulations (Leu, Neu, Ly, Mo, Eo, Ba); inflammatory markers (CRP, D-dimer, IL-6, RF, ASO, ECP); thyroid and metabolic parameters (TSH, FT4, PTH, vitamin D); immunoglobulins (IgG, IgA, IgM, IgE); complement components (C1 inhibitor concentration and activity, C3c, C4); autoantibodies (IgG anti-TPO, IgG anti-TG, IgG anti-TSH receptor, IgG anti-C1q, IgG anti-dsDNA, anticardiolipin IgG/IgM/IgA, ANA, ENA, pANCA, and cANCA); and tumor markers (CA 19-9, CEA, CA 15-3, CA 125).

### Detection of HSP70 and Anti-HSP70 antibodies

The measurement of HSP70 and anti-HSP70 antibodies was performed at a certified clinical laboratory.

HSP70 Quantification: Serum HSP70 levels were quantified using a high-sensitivity enzyme-linked immunosorbent assay (ELISA) kit (Enzo High Sensitivity HSP70 ELISA Kit, Catalog No. ADI-EKS-715) according to the manufacturer's instructions. Serum samples and standards were added to microplate wells pre-coated with a monoclonal antibody specific for human HSP70. After incubation and washing, a biotinylated detection antibody and streptavidin–horseradish peroxidase were applied. The reaction was developed using tetramethylbenzidine substrate, and absorbance was measured at 450 nm. HSP70 concentrations were calculated by interpolation from a standard curve generated using kit-provided standards.

Anti-HSP70 Antibody Detection: Total anti-HSP70 antibodies (IgG, IgA, and IgM) were measured using the Enzo Anti-HSP70 IgG/A/M ELISA Kit (Catalog No. ADI-EKS-750), following the manufacturer's protocol. Microplate wells coated with recombinant human HSP70 captured anti-HSP70 antibodies present in serum samples. After incubation and washing, an HRP-conjugated secondary antibody recognizing human IgG, IgA, and IgM was added. Absorbance was measured at 450 nm, and antibody concentrations were determined using a standard curve generated with kit-provided standards.

### Detection of IgG-anti-FcεRI and IgG-anti-IgE autoantibodies

Enzyme-linked immunosorbent assays (ELISA) were performed in 384-well MaxiSorp black plates (Thermo Fisher Scientific, Cat. No. 460518). All washing steps were conducted using phosphate-buffered saline (PBS) (pH 7.4) containing 0.05% Tween-20, 0.01% Triton X-100, and 0.5 M NaCl. Reagent diluent, which was also used for blocking, consisted of wash buffer supplemented with 10% non-protein blocking reagent (Li-Cor, Cat. No. LIC-927-90001).

IgG-anti-FcεRIα (IgG-aFcεRI): Plates were coated overnight at 4°C with 25 μl/well recombinant human FcεRIα (ACROBiosystems, Cat. No. FCA-H5228) at 1 μg/mL in PBS. After 3 washes, plates were blocked with 100 μl/well reagent diluent for 1 h at room temperature. Serum samples were diluted 1:100 in reagent diluent, and 25 μl/well of diluted serum or standard antibody (anti-human FcεRIα, Miltenyi Biotec, Cat. No. 130-122-308) was added and incubated for 1 h at room temperature. Following 3 additional washes, plates were incubated for 30 min with 25 μl/well HRP-conjugated goat anti-human IgG (Jackson ImmunoResearch, Cat. No. 109-035-098) diluted 1:40,000. After final washes, plates were developed with enhanced chemiluminescence (ECL) substrate and luminescence was measured immediately using a Victor plate reader.

IgG-anti-IgE (IgG-aIgE): For IgE-binding assays, plates were coated overnight at 4°C with 25 μl/well recombinant human myeloma IgE (Millipore/Sigma, Cat. No. 401152) diluted to 1 μg/mL in PBS. After washing and blocking as described above, serum samples were diluted 1:100 in reagent diluent, and recombinant anti-human IgE (clone REA1049, Miltenyi Biotec, Cat. No. 130-117-931) was used as standard. A total of 25 μl/well of diluted samples or standards was added and incubated for 1 h. Plates were washed and incubated with HRP-conjugated goat anti-human IgG (Jackson ImmunoResearch, Cat. No. 109-035-098) as described above, followed by ECL development and immediate luminescence measurement.

The ELISA assays used for the detection of IgG-aFcεRI and IgG-aIgE antibodies were applied for exploratory research purposes and do not have established clinical reference ranges or standardized diagnostic cut-off values. Therefore, antibody levels were analyzed as continuous variables within the study cohort, and patients were not categorized as antibody-positive or antibody-negative.

### Statistical analysis

Data distribution was assessed using the Shapiro–Wilk test. Continuous variables are presented as mean ± standard deviation (SD) for normally distributed data, or as median with the first and third quartiles (Q1–Q3) for non-normally distributed data. Categorical variables are expressed as absolute numbers and percentages.

For comparisons between groups with independent observations, one-way ANOVA and independent samples t-tests were used for normally distributed data. For non-normally distributed data, the Kruskal–Wallis H test and Mann–Whitney *U* test were applied. Associations between non-normally distributed continuous variables were evaluated using Kendall's Tau-b correlation.

Associations between IgG-aFcεRI and IgG-aIgE antibody levels and clinical or laboratory parameters were evaluated using the above statistical methods.

A significance level of p < 0.05 was considered statistically significant. Statistical analyses were performed using Jamovi (The Jamovi Project, version 2.7.5).

## Results

The study included 205 participants, predominantly female (77.6%), with a mean age of 42.8 years (range 18–87). Clinically, 38.8% of patients presented with urticaria only, 48.5% with urticaria and angioedema, and 12.6% with angioedema only. Detailed demographic and clinical characteristics of the study cohort are presented in Table 1.

**Table 1:** Demographic and clinical characteristics of the study cohort (n = 205)

Gender, n (%)	
Female	159 (77.6%)
Male	46 (22.4%)
Age	Mean (SD) 42.8 (15.2)
	Min./max. 18-87
Clinical features, n (%)	
Urticaria only	80 (38.8%)
Urticaria + angioedema	100 (48.5%)
Angioedema only	26 (12.6%)
Place of residence, n (%)	
City	181 (88.3%)
Rural area	24 (11.7%)
BMI (median, IQR)	25.5 (21.6–30.9)
Tobacco use, n (%)	
Non-smoker	113 (55.1%)
Smoker	59 (28.8%)
Former smoker	33 (16.1%)
Allergic comorbidity, n (%)	
No	135 (65.9%)
Yes	70 (34.1%)
Circadian symptom pattern, n (%)	
No circadian pattern	90 (43.9%)
Daytime	69 (33.7%)
Nighttime	46 (22.4%)
Accompanying symptoms, n (%)	
No	77 (37.6%)
Yes	128 (62.4%)
Disease-provoking factors, n (%)	
No	62 (30.2%)
Yes	143 (69.8%)
Therapy with antihistamines, n (%)	
No	14 (6.8%)
Yes	191 (93.2%)
Therapy with omalizumab, n (%)	
No	151 (73.7%)
Yes	54 (26.3%)
Therapy with cyclosporine, n (%)	
No	200 (97.6%)
Yes	5 (2.4%)

Patient-reported outcome measures (PROMs) describing disease activity, control, and quality of life are summarized in Table 2.

**Table 2:** Patient-reported outcome measures (PROMs): VAS, UCT, UAS7, CU-Q2oL, AAS7, ACT-3mo, and AE-QoL

Variable	n	Median (IQR)
VAS	139	8 (7–9)
UCT	139	8 (5.5–11)
UAS7	139	14 (4–28)
CU-Q2oL	139	34 (21–51)
AAS7	20	10.0 (0–78.8)
AECT-3mo	20	6.0 (3.8–9)
AE-QoL	19	38.2 (22.8–77.2)

Serum IgG-aFcεRI and IgG-aIgE levels were measured in 199 patients. The median IgG-aFcεRI level was 102.1 pg/mL (IQR 89.2–116.4), while the median IgG-aIgE level was 1760.8 pg/mL (IQR 1046.3–3742.5). Antibody levels showed considerable variability across the study cohort. Detailed laboratory findings are presented in Table 3.

**Table 3:** Laboratory findings

Variable	n	Median (IQR)
IgG-aFcεRI pg/mL	199	102.1 (89.20–116.40)
IgG-aIgE pg/mL	199	1760.8 (1046.25–3742.45)
Anti-HSP70 IgG/A/M pg/mL	176	1452 (1028.75–2157.5)
HSP70 pg/mL	174	0.0287 (0.00–0.15)
Leu × 10^9^/L	205	6.4 (5.35–7.59)
Neu × 10^9^/L	205	3.6 (2.74–4.40)
Ly × 10^9^/L	205	1.99 (1.70–2.40)
Mo × 10^9^/L	205	0.5 (0.40–0.62)
Eo × 10^9^/L	205	0.11 (0.09–0.20)
Ba × 10^9^/L	205	0.02 (0.00–0.05)
CRP mg/L	203	4 (4.00–4.00)
D-dim mg/L	117	0.33 (0.20–0.54)
D-vit ng/mL	169	31.9 (24.50–42.00)
TSH pg/mL	199	1.43 (1.09–2.16)
FT4 pmol/L	135	14.46 (12.87–16.15)
PTH pg/mL	59	38.1 (25.75–52.15)
IgG g/L	166	11.515 (10.13–12.78)
IgA g/L	165	2.17 (1.65–2.73)
IgM g/L	163	1.19 (0.83–1.63)
IgE IU/mL	191	89 (25.15–221.00)
C1 inh. Conc. G/L	52	0.3 (0.26–0.33)
C1 inh. act. %	62	111.8 (100.83–125.65)
C3c g/L	147	1.07 (0.92–1.26)
C4 g/L	159	0.23 (0.20–0.28)
dsDNS IU/mL	134	9.8 (7.38–9.80)
Anti-cardiolipin U/mL	91	3.3 (3.30–4.05)
IL-6 pg/mL	35	2.2 (2.00–4.15)
RF IU/mL	143	6.1 (3.50–8.00)
ASO IU/mL	83	101 (57.50–217.65)
ECP ng/mL	20	33.05 (21.53–50.83)
CA 19-9 U/mL	60	6.6 (3.50–12.98)
CEA ng/mL	74	0.6 (0.5–1.42)
CA 15-3 U/mL	47	11.2 (7.90–15.05)
CA 125 U/mL	64	11.5 (9.30–15.15)
	**n**	**%**
Anti-TPO negative	94	68.6
Anti-TPO positive	43	31.4
Anti-TSH negative	19	82.6
Anti-TSH positive	4	17.4
Anti-TG negative	48	80.0
Anti-TG positive	12	20.0
Anti-C1q Negative	44	86.3
Anti-C1q Positive	7	13.7
ANA negative	44	24.7
ANA positive	134	75.3
ENA negative	149	94.3
ENA positive	9	5.7
pANCA negative	132	97.1
pANCA positive	4	2.9
cANCA negative	135	99.3
cANCA positive	1	0.7

Comparisons between groups were performed using the Mann–Whitney *U* test and Kruskal–Wallis H test. Table 4, Table 5 present only statistically significant associations between antibody levels and clinical or laboratory variables. IgG-aFcεRI levels differed significantly according to anti-TPO status, with higher median levels in anti-TPO–positive patients (107 pg/mL, IQR 92.1–126) compared with anti-TPO–negative patients (97.7 pg/mL, IQR 87.9–111; p = 0.013).

**Table 4:** Comparison of IgG-aFcεRI and IgG-aIgE levels across patient subgroups.

IgG-aFcεRI						
Variable	Group	n	Median (IQR)	r	U	p
Anti-TPO	Neg	90	97.7 (87.9–111)	0.26852	1383	**0.013**
	Pos	42	107 (92.1–126)			
**IgG-aIgE**		**n**	**Median (IQR)**	**r**	**U**	**p**

Place of residence	City	173	1659 (1037–3624)	0.2575	1542	**0.041**
	Rural area	24	2206 (1759–3907)			
Accompanying symptoms	No	74	1528 (940–2898)	0.1936	3670	**0.023**
	Yes	123	2032 (1204–4312)			
Therapy with omalizumab	No	145	1614 (1011–2741)	0.2724	2743	**0.004**
	Yes	52	2531 (1534–5734)			

**Note:** Only statistically significant comparisons are shown

**Table 5:** Comparison of IgG-aFcεRI and IgG-aIgE levels across patient subgroups.

IgG-aFcεRI							
Variable	Group	n	Median (IQR)	X^2^	df	p	Ɛ^2^
Clinical features	Urticaria only	74	108 (98.1–121)	18.2	2	**< 0.001**	0.0922
	Urticaria + angioedema	98	95.3 (83.9–110)				
	Angioedema only	26	95.6 (85.6–106)				
**IgG-aIgE**							

		**n**	**Median (IQR)**	**X^2^**	**df**	**p**	**Ɛ^2^**
Circadian symptom pattern	No circadian pattern	107	1847 (1168–4326)	6.91	2	**0.032**	0.0353
	Daytime	58	1426 (903–3160)				
	Nighttime	32	2139 (1345–3130)				
Clinical features	Urticaria only	74	984 (852–1210)	82.3	2	**<**0**.001**	0.4178
	Urticaria + angioedema	98	2706 (1793–5840)				
	Angioedema only	26	1727 (1248–3835)				

**Note:** Only statistically significant comparisons are shown

For IgG-aIgE, significant differences were observed according to place of residence, presence of accompanying symptoms, and omalizumab therapy (Table 4). Median IgG-aIgE levels were higher in patients living in rural areas (2206 pg/mL, IQR 1759–3907) compared with patients living in urban areas (1659 pg/mL, IQR 1037–3624; Mann–Whitney *U* test, p = 0.041). Patients with accompanying symptoms had higher IgG-aIgE levels (2032 pg/mL, IQR 1204–4312) than those without accompanying symptoms (1528 pg/mL, IQR 940–2898; Mann–Whitney *U* test, p = 0.023). Patients receiving omalizumab therapy had higher IgG-aIgE levels (2531 pg/mL, IQR 1534–5734) compared with those not receiving omalizumab (1614 pg/mL, IQR 1011–2741; Mann–Whitney *U* test, p = 0.004).

Comparisons across more than 2 groups were evaluated using the Kruskal–Wallis H test (Table 5). IgG-aFcεRI levels differed significantly between clinical presentation groups (Kruskal–Wallis test, χ^2^ = 18.2, p < 0.001). Median levels were 108 pg/mL (IQR 98.1–121) in patients with urticaria only, 95.3 pg/mL (IQR 83.9–110) in patients with urticaria and angioedema, and 95.6 pg/mL (IQR 85.6–106) in patients with angioedema only.

IgG-aIgE levels also differed significantly between clinical presentation groups (Kruskal–Wallis test, χ^2^ = 82.3, p < 0.001). Median levels were 984 pg/mL (IQR 852–1210) in patients with urticaria only, 2706 pg/mL (IQR 1793–5840) in patients with urticaria and angioedema, and 1727 pg/mL (IQR 1248–3835) in patients with angioedema only.

IgG-aIgE levels differed between circadian symptom groups (Kruskal–Wallis test, χ^2^ = 6.91, p = 0.032). Median IgG-aIgE levels were 1847 pg/mL (IQR 1168–4326) in patients without a circadian symptom pattern, 1426 pg/mL (IQR 903–3160) in those reporting daytime symptoms, and 2139 pg/mL (IQR 1345–3130) in patients reporting nighttime symptoms (Table 5). No statistically significant differences in IgG-aFcεRI levels were observed across circadian symptom groups (Kruskal–Wallis test, p = 0.150).

Kendall's tau-b correlation analysis revealed several statistically significant associations (Table 6). IgG-aFcεRI levels showed a weak negative correlation with IgG-aIgE levels (r = −0.145, p = 0.003), neutrophil count (r = −0.107, p = 0.027), and total IgE (r = −0.153, p = 0.002), and a positive correlation with total IgG (r = 0.21, p < 0.001).

**Table 6:** Correlations of IgG-aFcεRI and IgG-aIgE levels with clinical and laboratory parameters

	IgG-aFceRI			IgG-aIgE		
Variable	n	r	p	n	r	p
Age	197	0,084	0,084	197	0,011	0,821
BMI	197	−0,033	0,494	197	0,024	0,617
IgG-aIgE	198	−0,145	**0,003**			
Anti-HSP70 IgG/A/M	171	0,063	0,225	171	−0,064	0,213
HSP70	169	−0,09	0,094	169	0,039	0,465
VAS	132	−0,094	0,136	132	0,156	**0,013**
UCT	132	0,02	0,742	132	−0,078	0,199
UAS7	132	0,031	0,609	132	0,047	0,432
CU-Q2oL	132	0,021	0,717	132	0,109	0,066
AAS7	20	−0,265	0,121	20	0,023	0,893
AECT-3mo	20	0,287	0,083	20	−0,027	0,87
AE-QoL	19	−0,212	0,207	19	0,094	0,575
Leu	197	−0,087	0,07	197	−0,068	0,16
Neu	197	−0,107	**0,027**	197	−0,06	0,209
Ly	197	−0,044	0,365	197	−0,069	0,157
Mo	197	−0,032	0,51	197	−0,067	0,172
Eo	197	−0,079	0,114	197	−0,034	0,494
Ba	197	−0,022	0,674	197	−0,041	0,426
CRO	195	0,008	0,888	195	−0,017	0,766
D-dim	111	0,041	0,532	111	0,079	0,225
D-vit	161	0,035	0,512	161	−0,037	0,49
TSH	191	0,088	0,073	191	−0,053	0,278
FT4	128	−0,008	0,892	128	−0,023	0,706
PTH	56	−0,033	0,724	56	−0,088	0,336
ASO	80	−0,098	0,199	80	0,092	0,23
ECP	20	−0,095	0,586	20	0,084	0,631
IgG	161	0,21	**<0,001**	161	0,049	0,356
IgA	160	−0,084	0,115	160	0,044	0,409
IgM	158	0,016	0,773	158	0,065	0,229
IgE	184	−0,153	**0,002**	184	0,053	0,289
C1 inh,	52	0,052	0,595	52	−0,164	0,094
C1 inh,funct,	62	0,084	0,337	62	−0,211	**0,016**
C3c	143	−0,015	0,796	143	−0,053	0,35
C4	155	0,054	0,331	155	−0,058	0,296
dsDNS	129	0,018	0,781	129	−0,048	0,454
Anti- cardiolipin	88	0,029	0,719	88	0,047	0,554
Il-6	34	−0,125	0,31	34	−0,031	0,8
RF	137	−0,031	0,612	137	0	1
CA 19-9	59	−0,056	0,53	59	0,063	0,48
CEA 5	73	0,021	0,8	73	−0,021	0,804
CA 15-3	46	−0,01	0,925	46	0,197	0,055
CA 125	63	−0,017	0,84	63	−0,118	0,174

IgG-aIgE levels showed a positive correlation with VAS scores (r = 0.156, p = 0.013) and a negative correlation with C1 inhibitor functional activity (r = −0.211, p = 0.016).

## Discussion

CSU symptoms are well established as significantly burdensome and capable of impairing quality of life depending on disease activity and severity. Numerous studies highlight the psychosocial and functional impact of CSU.^1^^,^^19^^,^^20^ In our cohort, despite most patients being under specialist care and receiving pharmacological treatment, PROMs indicated a persistent moderate-to-high disease burden (VAS 8 [IQR 7–9], UCT 8 [IQR 5.5–11], UAS7 14 [IQR 4–28], CU-Q2oL 34 [IQR 21–51]). Similarly, patients with angioedema reported notable disease impact (AAS7 10 [IQR 0–78.8], AECT-3mo 6 [IQR 3.75–9], AE-QoL 38.2 [IQR 22.8–77.2]). These findings are consistent with previous reports demonstrating that CSU remains inadequately controlled in a substantial proportion of patients despite treatment.^1^^,^^9^^,^^21^

Our study further explored associations between immunological markers and patient-reported symptom burden. IgG-aIgE levels showed a positive correlation with VAS scores, suggesting that higher levels of these autoantibodies may be linked to greater perceived symptom severity. Although direct studies examining IgG-aIgE in relation to VAS scores in CSU are limited, previous literature supports the association between autoantibody-mediated mechanisms and increased disease activity and severity.^22^, ^23^, ^24^, ^25^

We also observed higher IgG-aFcεRI levels in anti-TPO–positive patients. This finding is consistent with earlier reports linking type IIb autoimmune CSU with autoimmune thyroid disease.^8^^,^^12^ Previous studies suggest that the presence of anti-TPO antibodies, particularly in combination with low total IgE levels, may indicate a more pronounced autoimmune disease mechanism and a higher likelihood of antihistamine-refractory disease.^26^

In our cohort, IgG-aIgE levels differed according to place of residence, with higher levels observed among patients living in rural areas. Environmental factors have previously been proposed to influence CSU activity, including exposure to infections, allergens, or stressors.^27^ Differences in environmental exposures between rural and urban populations may therefore contribute to variation in immune responses and disease manifestations.^1^^,^^28^ However, the number of rural participants in our study was relatively small, and this finding should be interpreted with caution.

Distinct patterns of autoantibody distribution were observed across clinical phenotypes. Patients presenting with urticaria only showed higher IgG-aFcεRI levels, whereas IgG-aIgE levels were higher in patients with angioedema or combined urticaria-angioedema presentations. These findings suggest that different autoimmune pathways may contribute to distinct clinical manifestations of CSU.

We also found that patients reporting accompanying systemic symptoms had higher IgG-aIgE levels. Although limited literature directly addresses this observation, previous studies have suggested that elevated IgG-aIgE levels may be associated with more severe or treatment-refractory forms of CSU.^2^^,^^12^ Such findings raise the possibility that IgG-aIgE-mediated autoreactivity may be linked to broader systemic symptom profiles in some patients.

Patients with higher IgG-aIgE levels in our cohort were more frequently treated with omalizumab. Current treatment guidelines recommend omalizumab as the next therapeutic step for patients whose symptoms remain uncontrolled despite high-dose antihistamine therapy.^1^ Therefore, the observed association likely reflects disease severity and treatment escalation rather than a direct causal relationship between IgG-aIgE levels and omalizumab use.

An association between circadian symptom patterns and IgG-aIgE levels was also observed in our study. Patients reporting nighttime symptoms showed higher IgG-aIgE levels than those without circadian symptoms or with daytime symptoms. Although circadian influences on immune responses have been described in inflammatory conditions, studies specifically addressing circadian symptom patterns in CSU in relation to autoantibody profiles remain limited. To our knowledge, this relationship has not been previously investigated in CSU.

In our study, IgG-aFcεRI levels were higher in patients presenting with urticaria only compared with those with combined urticaria and angioedema or angioedema only, suggesting that FcεRI-targeting IgG autoantibodies may be more strongly associated with wheal-predominant CSU. Previous studies have also reported elevated IgG-aFcεRI levels in autoimmune CSU and associations with disease activity, although detailed comparisons between specific clinical phenotypes remain limited.^16^^,^^29^

In contrast, IgG-aIgE levels were higher in patients with angioedema only or in those with both urticaria and angioedema compared with patients presenting with urticaria alone. These findings suggest that different autoimmune pathways may contribute to distinct clinical manifestations of CSU, with FcεRI-directed autoimmunity potentially linked to wheal-predominant disease and IgE-targeting autoantibodies more relevant in angioedema-dominant or combined phenotypes. Importantly, this study did not assess markers of type I autoimmune CSU. Future studies should therefore evaluate both autoantibodies associated with type IIb autoimmunity and specific IgE antibodies to autoallergens to determine whether the associations observed here are clinically relevant and whether combined assessment of these markers may improve endotype identification in CSU.

A negative correlation was observed between IgG-aIgE levels and functional C1 inhibitor (C1-INH) activity. This finding raises the possibility that complement regulatory mechanisms may influence disease expression in a subset of CSU patients. Complement activation has previously been implicated in CSU pathogenesis, including reports of C4d deposition in lesional skin and increased serum C5a levels, suggesting activation of the classical complement pathway and potential involvement of immune complexes in inflammation.^30^ Dysregulation of complement and contact systems has also been proposed as an amplifying factor in severe or treatment-refractory CSU.^31^ However, the number of patients with available C1-INH functional measurements in our cohort was limited, and this observation should therefore be interpreted cautiously.

IgG-aFcεRI levels in our study demonstrated a distinct correlation pattern, showing negative correlations with IgG-aIgE, total IgE, and neutrophil count, while correlating positively with total IgG levels. The inverse relationship between IgG-aFcεRI and IgG-aIgE may indicate that these autoantibodies reflect different immunopathological endotypes of CSU. The positive correlation with total IgG may suggest broader immune activation, as increased immunoglobulin production has been reported in several autoimmune and inflammatory conditions.^32^, ^33^, ^34^, ^35^, ^36^ The relevance of these associations in CSU requires further investigation.

Although IgG-aFcεRI has previously been linked to autoimmune CSU, particularly in ASST-positive or treatment-resistant patients,^29^ its relationship with broader immune parameters remains incompletely understood. In our cohort, no significant associations were observed between IgG-aFcεRI, IgG-aIgE, and eosinophil or basophil counts, which have been proposed as potential biomarkers in CSU. For example, eosinopenia has been associated with high disease activity and type IIb autoimmune CSU, while basopenia has been reported in autoimmune subsets with poor treatment response.^12^^,^^29^^,^^37^^,^^38^ The absence of these associations in our study may reflect heterogeneity of patient populations or limitations of single-time-point laboratory measurements.

Additionally, several other laboratory markers did not reach statistical significance in our analyses, possibly due to limited sample sizes. These include parathyroid hormone (PTH), anti-streptolysin O (ASO), eosinophilic cationic protein (ECP), total C1-INH, interleukin-6 (IL-6), anti-thyroid peroxidase antibodies (anti-TPO), anti-C1q antibodies, and tumor markers such as CA 19-9, carcinoembryonic antigen (CEA), CA 15-3, and CA 125.

This study has several limitations. First, the autoantibody assays used in this study were applied for exploratory research purposes and do not have established clinical reference ranges or standardized diagnostic cut-off values. Therefore, antibody levels were analyzed as continuous variables rather than categorized as positive or negative. Second, the study did not include a healthy control group, which limits the ability to compare antibody levels with those in the general population. Third, some subgroup analyses involved relatively small numbers of patients, particularly those living in rural areas and those with available C1-INH functional measurements. In addition, several laboratory parameters were available only for subsets of patients (eg, total C1-INH, PTH, ASO, ECP, IL-6, IgG-anti-TPO, IgG-anti-C1q, CA 19-9, CEA, CA 15-3, and CA 125), which may have limited the statistical power of certain analyses. Finally, the cross-sectional design of the study does not allow conclusions about causal relationships between autoantibody levels and clinical manifestations of CSU.

Taken together, our findings support the concept that CSU represents a heterogeneous condition with multiple immunological mechanisms. Detailed clinical phenotyping, including symptom timing, presence of angioedema, and autoantibody profiles, may contribute to improved patient stratification and may support more individualized approaches to disease management.

## Conclusions

Our study suggests that elevated IgG-aIgE levels are associated with more severe clinical manifestations of CSU, including systemic symptoms, angioedema, and increased use of biologic therapy such as omalizumab. IgG-aIgE levels were also higher in patients from rural areas and showed novel associations with circadian nighttime symptom patterns. In contrast, higher IgG-aFcεRI levels were observed in patients with wheal-predominant disease and in those positive for anti-TPO. IgG-aIgE and IgG-aFcεRI levels demonstrated a negative correlation, which may reflect distinct but potentially complementary autoimmune pathways contributing to different clinical phenotypes of CSU. The absence of correlations between these autoantibodies and eosinophil or basophil counts in our cohort may reflect patient heterogeneity or limitations of single-time-point laboratory measurements, highlighting the complexity of CSU pathogenesis.

## Contribution

Lāsma Lapiņa**:** Conceptualization, Formal analysis, Investigation, Methodology, Project administration, Data collection, Resources, Formal analysis, Writing – review & editing.

Stefan Frischbutter: Investigation, Methodology, Writing – review & editing.

Jween Mohammad: Methodology, data collection, data analysis.

Guna Ziedone: Data collection, Writing – review & editing.

Nataļja Kurjāne: Conceptualization, Investigation, Methodology, Project administration, Data collection, Resources, Supervision, Writing – review & editing.

## Consent for publication

Not applicable.

## Availability of data and materials

All data generated or analyzed during this study are presented within this article. Additional information is available upon reasonable request by contacting the corresponding author.

## Ethics approval

Ethical approval for the study was granted by the Ethics Committee of Riga Stradiņš University (Nr. 2-PĒK-4/68/2023).

## Disclosure of the use of Generative AI and AI-assisted technologies

Generative artificial intelligence (AI) tools, specifically, ChatGPT (OpenAI), were used to assist with language editing and phrasing in this manuscript. No content, data interpretation, or scientific conclusions were generated or altered by AI.

## Funding

This work was supported by the doctoral grant “Internal and external consolidation of 10.13039/501100015042RSU in collaboration with LSPA”, No. 5.2.1.1. i.0/2/24/I/CFLA/005.

## Conflicts of interest

All authors declare no conflicts of interest in relation to this work.
